# Data supporting the use of end-tidal carbon dioxide (ETCO2) measurement to guide management of cardiac arrest: A systematic review

**DOI:** 10.1016/j.dib.2018.04.075

**Published:** 2018-04-25

**Authors:** Edison F. Paiva, James H. Paxton, Brian J. O’Neil

**Affiliations:** a University of São Paulo School of Medicine, Butantã, São Paulo 03178-200, Brazil; b Wayne State University School of Medicine, Detroit, MI 48201, USA

**Keywords:** Cardiac arrest, End tidal carbon dioxide, Prognostication, Advanced cardiac life support, Capnography, Systematic review, Meta-analysis

## Abstract

The data presented in this article are related to the research article, “The Use of End-Tidal Carbon Dioxide (ETCO_2_) Measurement to Guide Management of Cardiac Arrest: A Systematic Review” [1]. This article is a systematic review and meta-analysis of existing data on the subject of whether any level of end-tidal carbon dioxide (ETCO_2_) measured during cardiopulmonary resuscitation (CPR) correlates with return of spontaneous circulation (ROSC) or survival in adult patients experiencing cardiac arrest in any setting. These data are made publicly available to enable critical or extended analyses.

## Specifications Table

**Table:** 

Subject area	*Cardiac arrest, clinical evidence*
More specific subject area	*The utility of end-tidal carbon dioxide measurement as it correlates with return of spontaneous circulation (ROSC) or survival in adults experiencing cardiac arrest in any setting.*
Type of data	*Table, Figures*
How data was acquired	*Review of primary articles pertaining to end-tidal carbon dioxide measurement as it correlates with return of spontaneous circulation (ROSC) or survival in adults experiencing cardiac arrest in any setting.*
Data format	*Analyzed data*
Experimental factors	*Description of the published literature on end-tidal carbon dioxide measurement as it correlates with return of spontaneous circulation (ROSC) or survival in adults experiencing cardiac arrest in any setting.*
Experimental features	*Systematic review*
Data source location	*Original English-language articles identified from search of Embase, MEDLINE, and Cochrane Databases.*
Data accessibility	*Data are available with this article*

## Value of the data

• These data describe evidence available in the English-language medical literature pertaining to end-tidal carbon dioxide measurement as it correlates with return of spontaneous circulation (ROSC) or survival to hospital discharge in adult patients experiencing cardiac arrest in any setting.
• These data allow other researchers to extend the statistical analyses.

## 1 Data

**Fig. 1:**
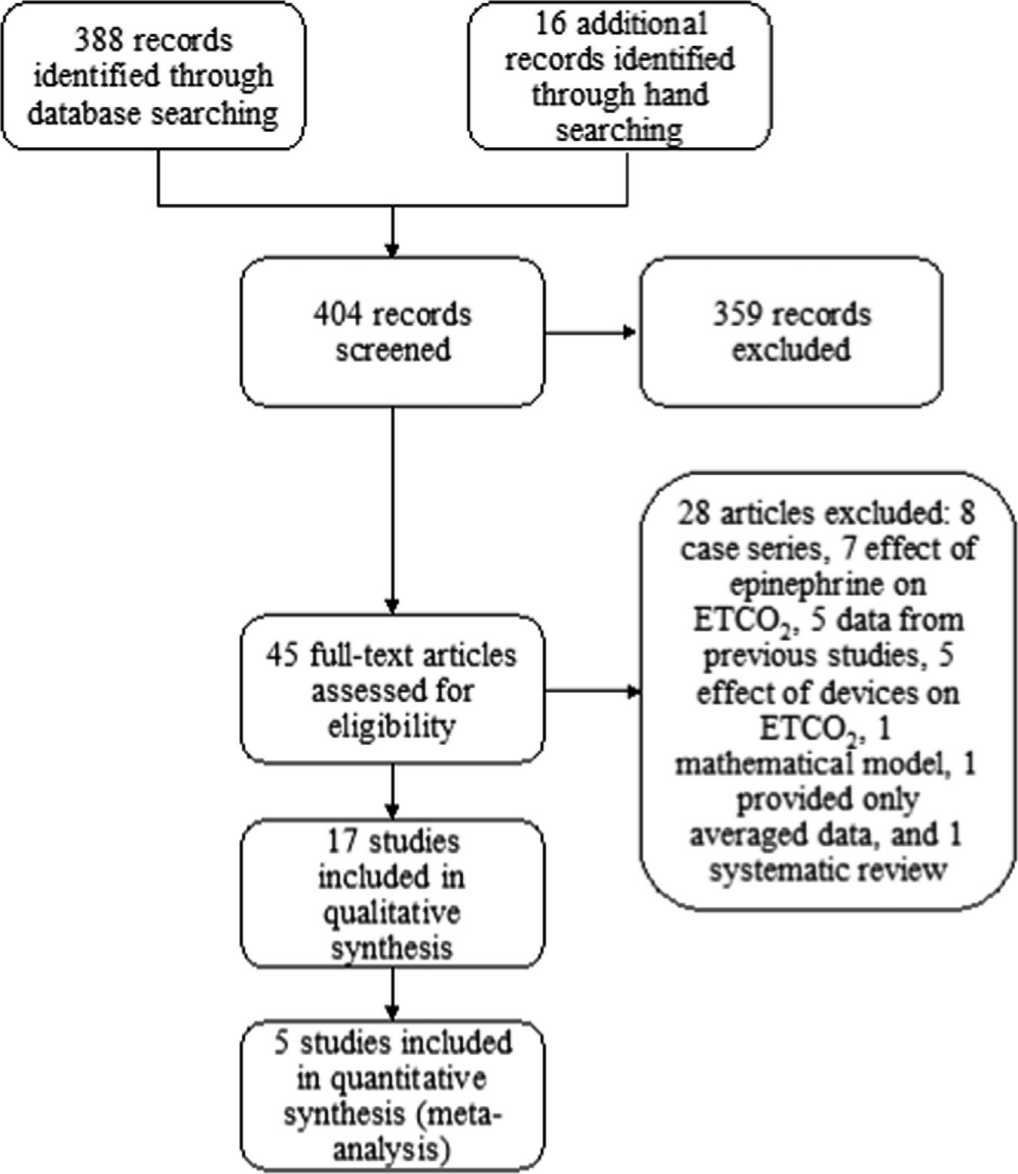
Flow diagram of search results.

**Fig. 2:**
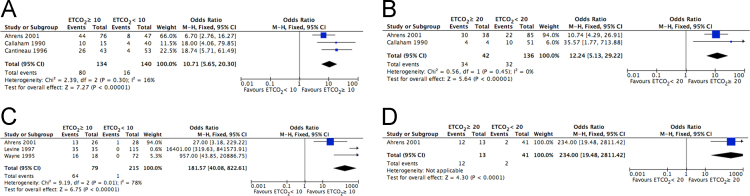
Forest plot of the correlation between ETCO_2_ and ROSC: A. Initial ETCO_2_ ≥ 10 mmHg; B. Initial ETCO_2_ ≥ 20 mmHg; C. 20-min ETCO_2_ ≥ 10 mmHg; D. 20-min ETCO_2_ ≥ 20 mmHg.

**Fig. 3:**
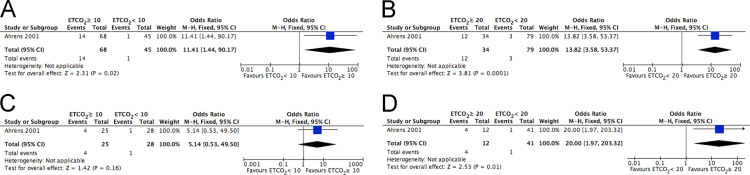
Forest plot of the correlation between specific ETCO_2_ levels and survival to hospital discharge: A. Initial ETCO_2_ ≥ 10 mmHg; B. Initial ETCO_2_ ≥ 20 mmHg; C. 20-min ETCO_2_ ≥ 10 mmHg; D. 20-min ETCO_2_ ≥ 20 mmHg.

These data report findings determined through the 2015 Consensus on Science and Treatment Recommendations process, managed by the International Liaison Committee on Resuscitation (www.ilcor.org/seers). These data include those studies that were considered to be most relevant in the determination of the utility of end-tidal carbon dioxide (ETCO2) measurement in the management of cardiac arrest in any setting. These data have been reported as the results of this effort to describe the use of ETCO2 in adult cardiac arrest, and are provided in a summary article describing their utility for adult patient experiencing cardiac arrest in any clinical setting [1]. A total of 17 full-text articles were included in the qualitative synthesis [2], [3], [4], [5], [6], [7], [8], [9], [10], [11], [12], [13], [14], [15], [16], [17], [18], and 5 articles were included in the quantitative analysis [3], [4], [5], [6], [7]. Fig. 1 shows a flow diagram of search results, including those full-text articles that were included in the qualitative synthesis and the quantitative analysis. Fig. 2 shows a Forest plot of the correlation between ETCO_2_ and ROSC. Fig. 3 shows a Forest plot of the correlation between specific ETCO_2_ levels and survival to hospital discharge. Table 1 shows the characteristics of the included studies. Table 2 shows a summary of findings, including ETCO_2_ level higher or lower than 10- or 20-mmHg for predicting outcome following cardiac arrest.

**Table 1:** Characteristics of the included studies.

**Study year**	**Design**	***N***	**Population**		**VF/VT (%)**	**Asystole/PEA (%)**	**ETCO**_**2**_**measurement**	**Time of ETCO**_**2**_**measurement (cut-off, mmHg)**	**Outcome(s)**	**Results**	**Potential bias**	**Included in meta-analysis**	**ROSC/Survival**
Ahrens 2001	Prospective cohort	127	IHCA and Helicopter	76.0		24.0^a^	Capnography	Initial, 5, 10, 15, 20 min, and final (≥ 10 and ≥ 20)	ROSC	ETCO_2_ ≥ 20 mmHg at 5 and 10 min – 94.4% of survival	Convenience sampling 14% have already achieved ROSC	Yes	43% ROSC
													31.5% STFH
									STFH	ETCO_2_ ≤ 17.5 mmHg at 15 min – 91.9% of non-survival			13.7% SHD
									SHD				
												
Callaham 1990	Prospective cohort	55	OHCA	10.9		54.5/34.6	Capnometry	Initial (≥ 10 and ≥ 20)	ROSC	ETCO_2_ ≥ 15 mmHg predicted ROSC (sensitivity 71% and specificity of 98%)	Rescuers not blinded	Yes	25.5% ROSC
											Small number of patients		

Cantineau 1996	Prospective cohort	120	OHCA	6.3		90.6/3.1	Capnometry	Initial and maximum (≥ 10)	ROSC	ETCO_2_ ≥ 10 mmHg predicted ROSC (sensitivity 87% and specificity of 74%)	90.6% asystole	Yes	31.7% ROSC
												
Wayne 1995	Prospective cohort	90	OHCA	0.0		0.0/100.0	Capnography	20 min (≥ 10)	ROSC	ETCO_2_ ≥ 10 mmHg predicted ROSC (sensitivity 97.3% and specificity 100.0%)	Only PEA	Yes	17.8% ROSC
									SHA				
													14.4% SHA
									SHD				
													7.8% SHD
												
Levine 1997	Prospective cohort	150	OHCA	0.0		0.0/100.0	Capnography	20 min (≥ 10)	ROSC	ETCO_2_ ≤ 10 mmHg predicted non-survival (sensitivity 100% and specificity of 100%)	Only PEA Includes data from Wayne's study	Yes	23.3% ROSC
									SHD				
													10.7% SHD
												
Sanders 1989	Prospective cohort	35	IHCA	47.3		27.1/25.6	Capnometry	Average (≥ 10)	ROSC	All patients with ROSC had average ETCO_2_ ≥ 10 mmHg	Small number of patients	No	25.7% ROSC
									SHD				
													8.6% SHD
												
Salen 2001	Prospective cohort	53	IHCA	0.0		0.0/100.0	Capnography	Initial (≥ 16)	SHA	ETCO_2_ ≥ 16 mmHg associated with survival to admission	Convenience sampling	No	11.3% SHA
											Small number of patients		

Eckstein 2011	Retrospective cohort	3121	OHCA	16.9		NA	Capnography	Initial (≥ 10 and ≥ 20)	ROSC	ETCO_2_ ≥ 10 mmHg associated with ROSC (OR 4.79; 95% CI 3.10 to 4.42)^c^	Retrospective large study, but an unreliable OR is provided	No	22.4% ROSC
Asplin 1995	Prospective cohort	27	OHCA	48.2		NA	Capnography	1 and 2 min (No specific cut-off)	ROSC	Higher ETCO_2_ levels in ROSC vs. non-ROSC (23.0 vs. 13.2 at 1 min, 26.8 vs. 15.4 at 2 min)	Convenience sampling	No	51.9% ROSC
									SHD				
											Small number of patients		
													11.1% SHD
												
Grmec 2001	Prospective cohort	139	OHCA	40.3		51.8/7.9	Capnometry	Initial, final and average (≥ 10)	ROSC	ETCO_2_ ≥ 10 mmHg predicted ROSC (sensitivity 100.0% and specificity of 74.1%, 81.4%, and 90.0%, respectively for initial, average, and final ETCO_2_)	–	No	38.1% ROSC
									SHD				
													16.6% SHD
												
Grmec 2003	Prospective cohort	185	OHCA	76.2		23.8^b^	Capnometry	Initial, final and average (≥ 10)	ROSC	Average and final ETCO_2_ higher in ROSC patients. Initial ETCO_2_ higher in ROSC patients only if cardiac origin	Includes data from Grmec 2001	No	64.3% ROSC
									SID				
													24.3% SID
												
Grmec 2007	Prospective cohort	389	OHCA	40.1		40.9/19.0	Capnometry	Initial, final and average (≥ 10)	ROSC	Initial ETCO_2_ ≥ 10 mmHg associated with ROSC	Includes data from Grmec 2003	No	60.9% ROSC
									SHA				
													50.1% SHA
									SHD				
													21.1% SHD
												
Heradstveit 2012	Retrospective cohort	575	OHCA	34.4		46.3/19.3	Capnography	Average, minimum and maximum (No specific cut-off)	ROSC	ETCO_2_ higher in ROSC patients	Retrospective	No	49.7% ROSC
									SHA				
													40.4% SHA
												
Kolar 2008	Retrospective cohort	737	OHCA	41.2		38.4/20.4	Capnometry	20 min (≥ 14.3)	ROSC	ETCO_2_ ≥ 14.3 mmHg predicted ROSC (sensitivity 100% and specificity 100%),	Retrospective Includes data from Grmec 2001, 2003, and 2007	No	59.4% ROSC
									SHA				
													54.6% SHA
									SHD				
													23.1% SHD
												
Lah 2011	Prospective cohort	114	OHCA	55.3		44.7^b^	Capnometry	Initial and every 1 min (No specific cut-off)	ROSC	Higher initial ETCO_2_ for those with ROSC if primary cardiac arrest (34.6 vs. 24.7 mmHg)	Comparison between asphyxial and cardiac origin of the arrest	No	63.2% ROSC
									SID				
													52.6% SID
									SHD				29.8% SHD
												
Mauer 1998	Prospective cohort	120	OHCA	49.1		17.9/33.0	Capnometry	Initial and every 2 min (≥ 15.0)	ROSC	All admitted patients had an ETCO_2_ ≥ 15 mmHg	ETCO_2_ was a secondary endpoint	No	57.5% ROSC
									SHA				
													27.5% SHA
									SHD				
													10.8% SHD
												
Rognås 2014	Prospective cohort	271	OHCA	NA		NA	Capnography	Initial (≥ 10)	ROSC	4/22 patients with ETCO_2_ ≤ 10 mmHg had ROSC.	23% lacking measurements	No	4 of 22 patients (18.2%) had ROSC with ETCO_2_ ≤ 1.3 kPa
										No specific cut-off should be used during resuscitation			

ETCO_2_, end-tidal CO_2_; NA, not available; IHCA, in-hospital cardiac arrest; OHCA, out-of-hospital cardiac arrest; OR, odds ratio; PEA, pulseless electrical activity; ROSC, return of spontaneous circulation; VF, ventricular fibrillation; VT, ventricular tachycardia; ROSC, return of spontaneous circulation; STFH, survival to twenty-four hours following cardiac arrest; SHA, survival to hospital admission; SID, survival to intensive care unit discharge; SHD, survival to hospital discharge.

a Includes asystole, PEA, and 14% in supraventricular tachycardia with a pulse, after intubation and first ETCO_2_ measurement.

b Includes asystole and PEA.

c Upper limit of confidence interval lower than OR.

**Table 2:** Summary of findings: ETCO_2_ higher vs. ETCO_2_ lower than 10 or 20 mmHg for predicting outcome following cardiac arrest.

**Quality assessment**							**No. of patients**		**Effect**		**Quality**	**Importance**
**No. of studies**	**Study design**	**Risk of bias**	**Inconsistency**	**Indirectness**	**Imprecision**	**Other considerations**	**ETCO**_**2**_**higher**	**ETCO**_**2**_**lower**	**Relative (95% CI)**	**Absolute (95% CI)**		

**ROSC (Initial ETCO**_**2**_** ≥ 10 vs. < 10 mmHg)**												
3^a^^,^^b^^,^^c^	observational studies	serious^d^	not serious	not serious	not serious	very strong association	80/134 (59.7%)	16/140 (11.4%)	OR 10.7 (5.6–20.3)	483 more per 1000 (from 326 more to 620 more)	LOW	CRITICAL
						dose response gradient						
**ROSC (Initial ETCO**_**2**_** ≥ 20 vs. < 20** **mmHg)**												
2^a^^,^^b^	observational studies	serious^d^^,^^e^	not serious	not serious	not serious	very strong association	34/42 (81.0%)	32/136 (23.5%)	OR 12.2 (5.1–29.2)	574 more per 1000 (from 406 more to 675 more)	LOW	CRITICAL
**ROSC (20 min ETCO**_**2**_** ≥ 10 vs. < 10 mmHg)**												
3^a^^,^^f^^,^^g^	observational studies	very serious^d^^,^^h^	serious^i^	not serious	not serious	very strong association	64/79 (81.0%)	1/215 (0.5%)	OR 181.6 (40.1–822.6)	805 more per 1000 (from 351 more to 966 more)	LOW	CRITICAL
						all plausible residual confounding would reduce the demonstrated effect						
**ROSC (20 min ETCO**_**2**_** ≥ 20 vs. < 20 mmHg)**												
1^a^	observational study	serious^d^	not serious	not serious	not serious	very strong association	12/13 (92.3%)	2/41 (4.9%)	OR 234,0 (19.5–2811.4)	874 more per 1000 (from 451 more to 944 more)	LOW	CRITICAL
**Survival at discharge (Initial ETCO**_**2**_** ≥ 10 vs. < 10 mmHg)**												
1^a^	observational study	serious^d^	not serious	not serious	not serious	very strong association	14/68 (20.6%)	1/45 (2.2%)	OR 11.4 (1.4–90.2)	184 more per 1000 (from 9 more to 650 more)	LOW	CRITICAL
**Survival at discharge (Initial ETCO**_**2**_** ≥ 20 vs. < 20 mmHg)**												
1^a^	observational study	serious^d^	not serious	not serious	not serious	very strong association	12/34 (35.3%)	3/79 (3.8%)	OR 13.8 (3.6–53.4)	315 more per 1000 (from 86 more to 640 more)	LOW	CRITICAL
**Survival at discharge (20 min ETCO**_**2**_** ≥ 10 vs. < 10 mmHg)**												
1^a^	observational study	serious^d^	not serious	not serious	serious^j^	none	4/25 (16.0%)	1/28 (3.6%)	OR 5.1 (0.5–49.5)	123 more per 1000 (from 18 fewer to 611 more)	VERY LOW	CRITICAL
**Survival at discharge (20 min ETCO**_**2**_** ≥ 20 vs. < 20 mmHg)**												
1^a^	observational study	serious^d^	not serious	not serious	not serious	very strong association	4/12 (33.3%)	1/41 (2.4%)	OR 20,0 (2.0–203.3)	309 more per 1000 (from 23 more to 811 more)	LOW	CRITICAL

All observational studies start with low quality ratings, and we have decided not to downgrade on risk of bias because of the very strong association between higher ETCO_2_ levels and ROSC or survival at discharge.

a Ahrens [3].

b Callaham [4].

c Cantineau [5].

d Convenience sampling, with 14% having already achieved ROSC.

e Small number of patients.

f Levine [7].

g Wayne [6].

h Includes data from previous study.

i high heterogeneity (*I*^2^ = 78%).

j Large confidence interval that crosses 1.0.

## 2 Experimental design, materials and methods

This review includes information on resuscitation questions developed through the 2015 Consensus on Science and Treatment Recommendations (CoSTR) development process, managed by the International Liaison Committee on Resuscitation (ILCOR) [19]. The questions were developed by ILCOR Task Force members, utilizing strict conflict of interest guidelines [20]. In general, each question was assigned to two experts to complete a detailed structured review of the literature, and complete a detailed evidence evaluation. Evidence evaluations are discussed at ILCOR meetings to reach consensus prior to publication as the Consensus on Science and Treatment Recommendations [19], [20], [21], [22].
